# Clinical outcome analysis of modified B-Lynch sutures in the fundus uteri and part of the corpus uteri for the prevention of intraoperative haemorrhage during caesarean delivery in women with twin pregnancy: a retrospective study

**DOI:** 10.1186/s12884-023-05759-3

**Published:** 2023-06-09

**Authors:** Chunbo Shi, Jinliang Chen, Aner Chen

**Affiliations:** 1 Department of Obstetrics and Gynecology, Ningbo Women and Children’s Hospital, Ningbo, 315012 Zhejiang China; 2 Radiology Department, Ningbo Women and Children’s Hospital, Ningbo, 315012 Zhejiang China

**Keywords:** Modified B-Lynch, Twin pregnancy, Caesarean delivery, Uterine atony

## Abstract

**Objective:**

To explore the efficacy of modified B-Lynch sutures in the fundus uteri and part of the corpus uteri for the prevention of intraoperative haemorrhage during caesarean delivery in women with twin pregnancy.

**Methods:**

This retrospective analysis covers the clinical data of 40 women with postpartum haemorrhage caused by uterine inertia during caesarean section in women with twin pregnancy in our hospital from January 2018 to May 2022. These women were divided into the group with modified B-Lynch sutures at the fundus and part of the corpus uteri (Group A, 20 patients) and the group with classic B-Lynch sutures (Group B, 20 patients) according to the treatment received. The treatment effect and safety of the two uterine compression sutures were compared.

**Results:**

In this study, no statistically significant differences were found in the outcomes of haemostasis or intraoperative and 24-h postoperative blood loss between the two uterine compression suture groups (*P* > 0.05). Compared to Group B, Group A showed a significantly reduced operative time, postoperative hospital stay, puerperal morbidity rate, pain score and duration of lochia.

**Conclusion:**

Modified B-Lynch sutures at the fundus and part of the corpus uteri can achieve a haemostatic effect similar to that of the classic B-Lynch suture, while it allows for a shortened operative time and reduced postoperative complications. Modified B-Lynch sutures can serve as a safe, rapid and effective haemostatic method for the prevention and treatment of postpartum haemorrhage during caesarean section in women with twin pregnancy, showing certain validity for promotion in clinics.

## Introduction

The increasing development of assisted reproductive technology promotes the incidence of twin pregnancy, in which the uterine fibres will be overstretched, resulting in difficulty in uterine fibre contraction and retraction after delivery, that is, uterine atony. As a result, the risk of postpartum haemorrhage in women with twin pregnancy is significantly increased and is reported to be 2. 26 to 4 times higher than that of women with singleton pregnancy according to some studies [1–3]; additionally, the risk of severe postpartum haemorrhage is reported to be 2.11 times higher [4]. Caesarean delivery for twin pregnancy is a difficult kind of caesarean section [5]. Currently, postpartum haemorrhage remains the leading cause of maternal mortality worldwide [6], of which 70% to 80% of cases occur due to weak uterine contractions, significantly increasing the difficulty of treating postpartum haemorrhage [7]. At present, the most common surgical procedures applied to treat postpartum haemorrhage due to uterine inertia refer to various methods derived from the B-Lynch uterine compression suture. Considering the limited data to date, we cannot draw a conclusion about the superiority of any available suturing technique. The most appropriate uterine compression suture for each patient should be determined based on the site and the cause of uterine bleeding, and also depends on the experience of the surgeon. Over the years, we have devoted ourselves to studying the prevention and treatment of postpartum haemorrhage during caesarean section in women with twin pregnancy and have promoted a new uterine compression suture method, that is, the modified B-Lynch compression suture for the fundus and part of the corpus uteri; this method additionally allows for a narrowed scope of compression that involves only the specific areas in the fundus and part of the corpus uteri where uterine atony occurs. In this retrospective study, 40 women with twin pregnancy and postpartum haemorrhage caused by uterine inertia during caesarean section in our hospital from January 2018 to May 2022 were enrolled to compare the treatment effect and safety of the two uterine compression sutures.

## Materials and methods

The retrospective analysis covered the clinical data of 40 women with twin pregnancy and postpartum haemorrhage caused by uterine inertia during caesarean section in our hospital from January 2018 to May 2022. Participants were divided into the group with modified B-Lynch sutures at the fundus and part of the corpus uteri (Group A) and the group with classic B-Lynch sutures (Group B) according to the treatment received. Group A involved 20 patients, including 3 multiparas, 17 primiparas, and 2 patients with uterine scars, with a mean maternal age of 32.2 ± 5.5 years and a mean gestational week of 35.7 ± 1.0 weeks. Group B included the other 20 patients, including 5 multiparas, 15 primiparas, and 4 patients with uterine scars, with a mean maternal age of 32.3 ± 4.2 years and a mean gestational week of 35.8 ± 1.3 weeks. Inclusion criteria were as follows: patients with twin pregnancy who underwent caesarean section, patients with postpartum haemorrhage caused by uterine inertia, and patients with unsatisfactory uterotonic effects. Exclusion criteria were as follows: (1) Patients with a history of underlying coagulation dysfunction or haemopathy and (2) Patients with unstable vital signs or combined disseminated intravascular coagulation(DIC). There was no significant difference in age, gravidity, parity, gestational week or other general clinical data between the 2 groups (*P* > 0.05) (Table 1). As this was a retrospective study and the operation was performed with the consent of the patient or her immediate family, ethical approval was not needed.

**Table1:** Baseline characteristics of patients

	**Group A(*n* = 20)**	**Group B(*n* = 20)**	**P**
Age (year)	32.2 ± 5.5	32.3 ± 4.2	> 0.05
Gravidity	1.5(1,2)	2(1,3)	> 0.05
Parity	0(0,0)	0(0,0.75)	> 0.05
Gestational age(week)	35.7 ± 1.0	35.8 ± 1.3	> 0.05
Previous CS n(%)	2(10)	4(20)	> 0.05
Combined birthweight (g)	4965 ± 468	4876 ± 540	> 0.05

### Group A

**Fig. 1:**
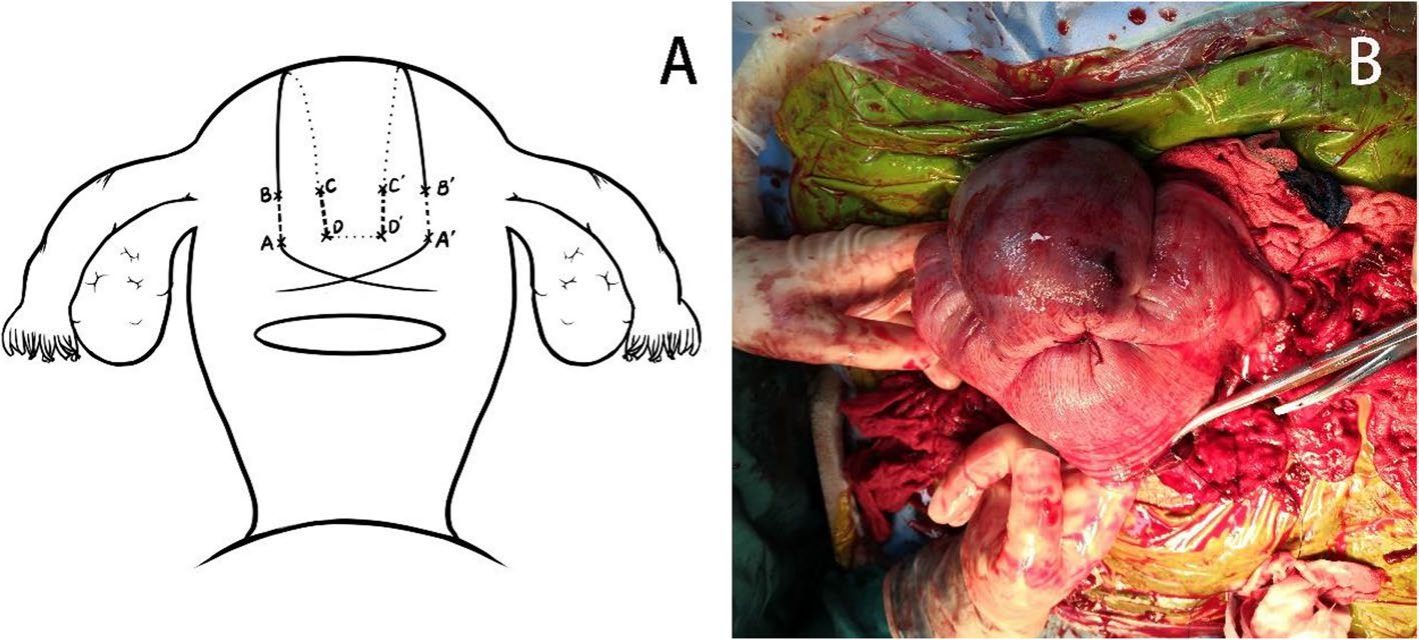
**A** Steps of modified B-Lynch sutures in the fundus uteri and part of the corpus uteri; the letters represent the puncture point. **B** Positive-anterior view of modified B-Lynch sutures

After the uterus was exteriorized, the extent of uterine atony was determined. Then, bimanual compression was applied in the range. If bleeding stopped with compression, we performed local compression suturing within this range. The suture material was a 75-cm atraumatic number 1 chromic catgut with a 40-mm round needle. The suture began at the right inferior edge of the range. The suture was placed vertically in the anterior wall of the uterus at a depth of 1–2 cm to prevent puncturing the endometrium (A → B). The suture was then fed anteriorly and vertically over the fundus and then passed back, posteriorly and downwards, to enter the posterior wall of the uterus at the same level as the upper anterior exit point. The suture was placed vertically from top to bottom at a depth of 1–2 cm to prevent puncturing the endometrium (C → D). The needle was passed in the same fashion into the left side through the posterior wall of the uterus and out (D ‘ → C’).The suture was fed posteriorly and vertically over the fundus to lie anteriorly and vertically over the fundus on the left side as performed on the right side. The needle was passed in the same fashion into the left side and through the anterior wall of the uterus (B ‘ → A’). The 2 ends of the suture were tied with a double throw knot to maintain tension (see Fig. 1).

### Group B

**Fig. 2:**
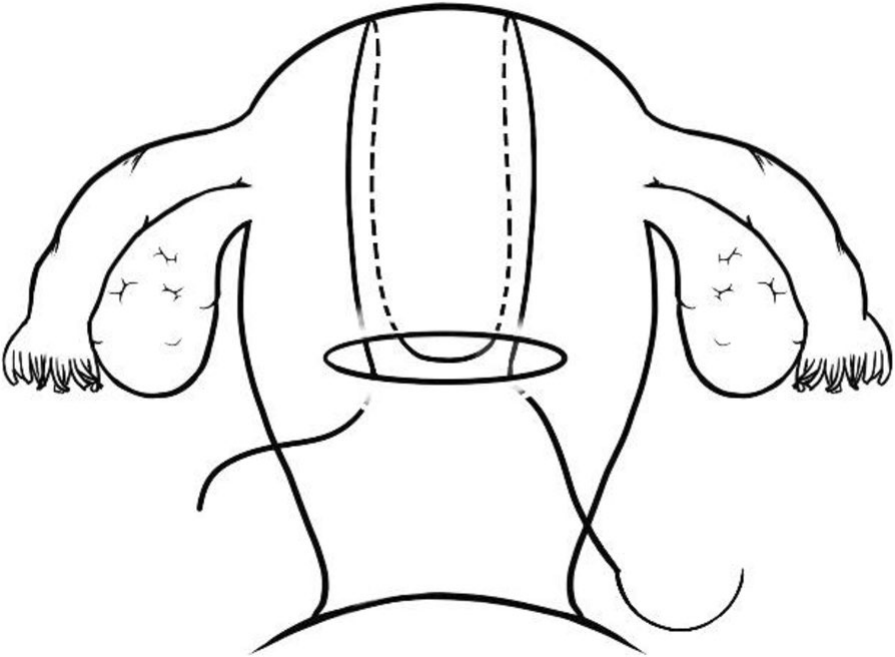
Steps of the classic B-Lynch sutures

Classic B-lynch sutures were given according to Mallagappa's method [8]. With the bladder displaced inferiorly, a No. 1 Polysorb CL-905 suture on a 70-mm round-bodied hand-held needle was placed in the uterus 3 cm below the right lower edge of the uterine incision and 3 cm from the right lateral border of the uterus. The suture was then threaded through the uterine cavity and emerged at the upper incision margin 3 cm above and approximately 4 cm from the lateral border. The suture was passed over to compress the uterine fundus approximately 3–4 cm from the right cornual border. It was then fed posteriorly and vertically to enter the posterior wall of the uterine cavity at the same level as the upper anterior entry point. The suture was pulled under moderate tension assisted by manual compression exerted by the Wrst assistant and then passed back posteriorly in a horizontal direction through the same surface marking as that on the right side. Then, it was fed posteriorly and vertically over the fundus to lie anteriorly and vertically, compressing the fundus on the left side, as performed on the right side. The needle was passed in the same fashion on the left side: through the uterine cavity and out approximately 3 cm anterior to and below the lower incision margin on the left side. The 2 ends of the suture were tied with a double throw knot to maintain tension (see Fig. 2).

Following the delivery of the newborn and placenta, all the women were injected intravenously with 0.1 mg carbetocin. In all women, there was no significant bleeding from the fundus or uterine body after receiving a modified B-Lynch suture in the fundus and part of the uterine body. In patients with lower uterine bleeding, a combination of compression of the lower uterine body by balloon tamponade or ligation of the uterine artery was required to reduce the uterine blood supply to effectively control the lower uterine bleeding.

All patients underwent rechecks with uterine ultrasound 42 days after surgery to determine whether intrauterine adhesions were present and were seen for follow-up visits from 6–12 months, which included the period of lochia, the recovery time of menstruation, the number of menstrual cycles, and whether they had become pregnant again.

Blood loss during the operation (ml) was calculated as the amount of blood collected in the blood container + the number of dressings × the difference in weight before and after gauze soaking × 1.05; blood loss after the operation (ml) was calculated as the difference in mass before and after puerperal pad soaking × 1.05. Haemostatic efficacy was defined as follows [9]: vaginal bleeding volume < 50 mL/h, good uterine contractions, stable vital signs and a normal urine output indicated that the operation was effective; a vaginal bleeding volume > 50 mL/h, poor uterine contractions, unstable vital signs, a urine output < 30 mL/h or no urine output indicated that the operation was ineffective, and hysterectomy was required.

Statistical analysis was performed using SPSS 23.0 software. Data are presented as the mean ± SD, median (P25, P75) or counts (percentages). Comparisons between groups were determined by t tests, Wilcoxon rank sum tests or chi-square tests. *p* < 0.05 was considered significant.

## Results

The comparison of outcomes between the two groups of patients is summarized in Table 2. The two uterine compression sutures demonstrated similar haemostasis outcomes, without significant differences in the rates of uterine balloon tamponade, uterine artery ligation and uterus retention (*P* > 0.05).

**Table 2:** The comparison of outcomes between the two groups of patients

	**Group A(*n* = 20)**	**Group B(*n* = 20)**	**P**
Age (year)	32.2 ± 5.5	32.3 ± 4.2	> 0.05
Gravidity	1.5(1,2)	2(1,3)	> 0.05
Parity	0(0,0)	0(0,0.75)	> 0.05
Gestational age(week)	35.7 ± 1.0	35.8 ± 1.3	> 0.05
Previous CS n(%)	2(10)	4(20)	> 0.05
Combined birthweight (g)	4965 ± 468	4876 ± 540	> 0.05

Table 3 shows a summary of the comparison of the operation time, bleeding volume, and blood transfusion volume between the two groups of patients. Group A showed a significantly reduced operative time compared to Group B (*P* < 0.05). No statistically significant differences were found in the intraoperative and 24-h postoperative blood loss and blood transfusion volumes between the two groups (*P* > 0.05).

**Table 3:** The comparison of the operation time, bleeding volume, and blood transfusion volume between the two groups of patients

	**Group A(*n* = 20)**	**Group B(*n* = 20)**	**P**
Operation time (min)	73.6 ± 14.7	83.3 ± 14.9	0.045
Intraoperative blood loss (ml)	960 ± 266	985 ± 372	> 0.05
24-h postoperative blood loss(ml)	189 ± 39	215 ± 52	> 0.05
Transfusion n(%)	3(15)	4(20)	> 0.05

The postoperative recovery outcomes between the two groups of patients is summarized in Table 4. Group A showed a significantly reduced postoperative hospital stay, puerperal morbidity rate, pain score and duration of lochia compared to Group B(*P* < 0.05). Due to the short period of follow-up, all patients took precautions without plans for further pregnancies.

**Table 4:** The postoperative recovery outcomes between the two groups of patients

	**Group A(*n* = 20)**	**Group B(*n* = 20)**	**P**
Postoperative hospital stay (d)	4 ± 0.6	5 ± 0.6	< 0.001
Puerperal morbidity n(%)	1(5)	6(30)	0.037
VAS score	3.6 ± 0.9	4.2 ± 0.95	0.046
Pyometra n(%)	0(0)	1(5)	> 0.05
Duration of lochia (d)	35.5 ± 4.2	38.1 ± 3.5	0.039
Resumed menstruation (m)	5.2 ± 1.4	5.1 ± 1.0	> 0.05
Intrauterine adhesion	0(0)	0(0)	> 0.05
Change in menstrual flow n(%)			> 0.05
Increase	3(15)	2(10)	
Reduction	4(20)	5(25)	
No change	13(65)	13(65)	

## Discussion

Postpartum haemorrhage (PPH) is more likely in twin pregnancy than in singleton pregnancy [10]. The overdilation of the uterus affects the contractility of the myometrium after delivery and leads to uterine atony, which could be the main source of postpartum haemorrhage in twin pregnancy. In twin pregnancy, the maternal blood volume and uterine blood flow is increased to maintain placental circulation and foetal growth and development, which could also elevate the risk of postpartum haemorrhage [11]. The primary measures for postpartum haemorrhage in women with twin pregnancy undergoing caesarean section refer to massaging the uterus and uterotonics. Oxytocin, methergine, and prostaglandin are adopted as common uterotonics, and there is no existing evidence regarding which one is the most effective [12]. When a uterotonic does not work, one or more surgical methods are required to stop the bleeding, including uterine compression sutures, uterine packing, pelvic vessel ligation, and transcatheter arterial embolization. Subtotal hysterectomy or total hysterectomy should be performed when a mother’s life is at risk and aggressive resuscitation does not work [13, 14]. Due to its simplicity and cost-effectiveness, the B-Lynch suture has been adopted as the preferential method for postpartum haemorrhage [15], which has contributed to multiple cured refractory postpartum haemorrhages and prevented hysterectomy since it was first introduced by Dr. B-Lynch in 1997 [16]. In the last 25 years, various derivations of the B-Lynch uterine compression suture have been successively reported [17]. However, several long-term complications of B-lynch sutures have been reported in recent literature, involving myometrial damage and necrosis, uterine rupture in mid to late pregnancy, intrauterine adhesions, abnormal menstruation, and secondary infertility, which may be related to excessive uterine compression and uterine cavity penetration by sutures [18–20].

The modified B-Lynch suture of the fundus and part of the corpus uteri was developed from a modification of the classic B-Lynch suture, which can effectively compress the arcuate vessels of the uterine wall by mechanical longitudinal compression of the smooth muscle of the fundus and corpus uteri, leading to reduced and moderated blood flow that forms local haemostasis and thrombosis. As a result, the myometrium experiences ischaemia, thus stimulating the uterus to contract and compressing the blood sinus to stop the haemorrhage [21]. In addition, the suture exerts continuous pressure and intensifies contractions on the fundus and part of the corpus uteri, and the polar conduction of contractions produces surface-to-deep compression of the lower uterine segment to achieve continuous uterine contraction, thus alleviating uterine asthenia [22]. This suture is similar to the classic B-Lynch suture in the primary principle of haemostasis, and this study also demonstrated similar outcomes of haemostasis by both methods, without significant differences in intraoperative and 24-h postoperative blood loss and blood transfusion volumes. These two methods are more effective for single uterine bleeding, and uterine atony throughout the uterus, including the lower uterine segment, resulting from twin foetuses requires combination with other haemostatic measures to achieve the desired haemostatic effect, in which uterine artery ligation and uterine balloon tamponade are commonly utilized. In this study, no statistically significant differences were found in the rates of uterine balloon tamponade and uterine artery ligation between the two groups.

The modified B-Lynch suture of the fundus and part of the corpus uteri has unique advantages in the following aspects. First, the "core" lies the compression suture, which is limited in its placement [23]. The extent of uterine compression is adjusted according to the extent of uterine atony, which makes the modified B-Lynch suture more specific and flexible and easier to perform, reducing compression damage to the myometrium and lowering the incidence of postoperative complications including uterine necrosis and uterine rupture. Second, the decidua is not penetrated with this approach, leading to the preserved integrity of the uterine cavity and endometrium and the reduced incidence of intrauterine adhesions, uterine mucosal necrosis, and bleeding [24, 25]. Furthermore, this approach allows for a shorter distance of the sutures wrapped around the anterior and posterior walls of the uterus, and one mattress suture is made in both the anterior and posterior wall to ensure the immobility of sutures to prevent obstruction due to the possibility of wire loops penetrating other organs. Theoretically, modified B-Lynch sutures in the fundus and part of the corpus uteri can alleviate the long-term complications associated with classic B-Lynch sutures and improve the prognosis.

In this study, Group A showed a significantly reduced operative time, postoperative hospital stay, puerperal morbidity rate, and pain score compared to Group B. The convenience of using modified B-Lynch sutures at the fundus and part of the corpus uteri could lead to a significantly shortened operation time and reduced damage to the myometrium, so postoperative uterine tenderness is significantly reduced, accompanied by a decreased number of postpartum fever cases and reduced postoperative hospital stays, puerperal morbidity rates and pain scores. The duration of lochia in Group A was significantly reduced compared to that in Group B, which could be explained by the sutures not penetrating the uterine cavity, so the endometrium was preserved. Although no significant difference was found in the incidence of long-term postoperative complications between the two groups, one patient in Group B who received an intraoperative classic B-Lynch suture and underwent bilateral ascending uterine artery ligation experienced infection and suppuration of the uterine incision 10 days after surgery, which was cured by anti-infection measures and ultrasound-guided puncture. The reason for this might be ischaemic necrosis of the uterine incision caused by excessive compression with the classic B-Lynch suture and uterine artery ligation.

## Conclusion

In conclusion, modified B-Lynch sutures at the fundus and part of the corpus uteri can achieve a haemostatic effect similar to that of classic B-Lynch sutures, while they allow for a shortened operative time and reduced postoperative complications. Modified B-Lynch sutures can serve as a safe, rapid and effective haemostatic method for the prevention and treatment of postpartum haemorrhage during caesarean section in women with twin pregnancy, showing certain validity for promotion in clinics. The shortcomings of this study are that the present sample was limited, and the effect on long-term recovery and subsequent pregnancy requires further study by increasing the follow-up time.

## Data Availability

The research data used to support the fndings of this study were supplied by Ms. Shi under license and so cannot be made freely available. Requests for access to these data should be made to Ms. Shi (Email:15824506880@ 163.com).

## Abbreviations

DIC: Disseminated intravascular coagulation
PPH: Postpartum haemorrhage

## References

[1] Blitz MJ, Yukhayev A (2020). Twin pregnancy and risk of postpartum hemorrhage. Matern Fetal Neonatal Med.

[2] Suzuki S, Inde Y, Igarashi M (2008). Elective cesarean as a risk factor for transfusion after delivery of twins. Nippon Med Sch.

[3] Sentilhes L, Merlot B, Madar H (2016). Postpartum haemorrhage: prevention and treatment. Expert Rev Hematol.

[4] Magann EF, Evans S, Hutchinson M (2005). Postpartum hemorrhage after cesarean delivery: an analysis of risk factors. South Med J.

[5] Visconti F, Quaresima P, Rania E (2020). Difficult caesarean section: A literature review. Eur J Obstet Gynecol Reprod Biol.

[6] Say L, Chou D, Gemmill A (2014). Global causes of maternal death: a WHO systematic analysis. Lancet Glob Health.

[7] Luo F, Chen M, Zhang Li (2012). Comparison of the efficacy of five haemostatic procedures for refractory postpartum haemorrhage and analysis of the causes of haemostatic failure. Chin J Obstet Gynecol.

[8] Mallappa Saroja CS, Nankani A, El-Hamamy E (2010). Uterine compression sutures, an update: review of efficacy, safety and complications of B-Lynch suture and other uterine compression techniques for postpartum haemorrhage. Arch Gynecol Obstet.

[9] Sentilhes L, Vayssière C, Mercier FJ (2014). Postpartum hemorrhage: Guidelines for clinical practice - Text of the Guidelines (short text). Gynecol Obstet Biol Reprod (Paris)..

[10] Kramer MS, Berg C, Abenhaim H (2013). Incidence, risk factors, and temporal trends in severe postpartum hemorrhage. Am J Obstet Gynecol.

[11] Owiredu WKBA, Osakunor DNM, Turpin CA (2016). Laboratory prediction of primary postpartum haemorrhage: a comparative cohort study. BMC Pregnancy Childbirth.

[12] Pacheco LD, Saade GR, Hankins GDV (2019). Medical management of postpartum hemorrhage: An update. Semin Perinatol.

[13] Shakur-Still H, Roberts I (2018). Finding Better Ways to Prevent Postpartum Hemorrhage. N Engl J Med.

[14] Santana DS, Cecatti JG (2016). Twin Pregnancy and Severe Maternal Outcomes: The World Health Organization Multicountry Survey on Maternal and Newborn Health[J]. Obstet Gynecol.

[15] Sun X, Huang Y, Zhang H (2016). B-Lynch suture in the treatment of postpartum hemorrhage due to uterine atony and impact on long-term fertility. Chin J Perinatal Med..

[16] B-Lynch C, Coker A, Lawal AH (1997). The B-Lynch surgical technique for the control of massive postpartum haemorrhage: an alternative to hysterectomy? Five cases reported. Br J Obstet Gynaecol.

[17] Matsubara S, Yano H, Ohkuchi A (2013). Uterine compression sutures for postpartum hemorrhage: an overview. Acta Obstet Gynecol Scand.

[18] Jiang L, Yang F (2019). A case report of complications following a combination of modulated B-lynch and Hwu sutures in postpartum haemorrhage: haematocele in the uterine cavity, hemoperitoneum and swelling and rupture of the fallopian tube. Obstet Gynaecol.

[19] Sathe NA, Likis FE, Young JL (2016). Procedures and Uterine-Sparing Surgeries for Managing Postpartum Hemorrhage: a systematic review. Obstet Gynecol Surv.

[20] Arab M, Ghavami B, Saraeian S, et al,. Successful Management of Two Cases of Placenta Accreta and a Literature Review: Use of the B-Lynch Suture and Bilateral Uterine Artery Ligation Procedures. Iran Red Crescent Med J. 2016, 9;18(4):e35006. 10.5812/ircmj.35006 PMC4921213 27354921

[21] Lynch B, Einspruch EL (2010). With or without an instructor, brief exposure to CPR training produces significant attitude change. Resuscitation..

[22] Wen R (2019). Clinical study on the treatment of postpartum haemorrhage by hand-held compression of the lower uterine segment. Contemp Med.

[23] Hao YING (2011). Uterine Compression Sutures: Yesterday, Today and Tomorrow. J Int Obstet Gynecol.

[24] Mascarello KC, Horta BL, Silveira MF (2017). Maternal complications and cesarean section without indication: systematic review and meta-analysis. Rev Saude Publica.

[25] Şahin H, Soylu Karapınar O, Şahin EA (2018). The effectiveness of the double B-lynch suture as a modification in the treatment of intractable postpartum haemorrhage. Obstet Gynaecol.

